# Phytoagent deoxyelephantopin derivative inhibits triple negative breast cancer cell activity by inducing oxidative stress-mediated paraptosis-like cell death

**DOI:** 10.18632/oncotarget.18183

**Published:** 2017-05-25

**Authors:** Jeng-Yuan Shiau, Kyoko Nakagawa-Goto, Kuo-Hsiung Lee, Lie-Fen Shyur

**Affiliations:** ^1^ Institute of Biotechnology, National Taiwan University, Taipei, Taiwan; ^2^ College of Medical, Pharmaceutical and Health Sciences, Kanazawa University, Kanazawa, Japan; ^3^ Natural Products Research Laboratories, Eshelman School of Pharmacy, University of North Carolina, Chapel Hill, North Carolina, USA; ^4^ Agricultural Biotechnology Research Center, Academia Sinica, Taipei, Taiwan; ^5^ PhD Program in Translational Medicine, College of Medicine, Kaohsiung Medical University, Kaohsiung, Taiwan

**Keywords:** breast cancer, sesquiterpene lactone, oxidative stress, paraptosis

## Abstract

Triple negative breast cancer (TNBC) is a highly metastatic cancer among the breast cancer subgroups. A thorny issue for clinical therapy of TNBC is lack of an efficient targeted therapeutic strategy. We previously created a novel sesquiterpene lactone analog (named DETD-35) derived from plant deoxyelephantopin (DET) which exhibits potent effects against human TNBC MDA-MB-231 tumor growth in a xenograft mouse model. Here we studied the mechanisms of both DET and DETD-35 against MDA-MB-231 cells. DETD-35 (3-fold decreased in IC_50_) exhibited better anti-TNBC cell activity than DET as observed through induction of reactive oxygen species production (within 2 h treatment) and damage to the ER structures, resulting in ER-derived cytoplasmic vacuolation and ubiquitinated protein accumulation in the treated cells. Intriguingly, the effects of both compounds were blockaded by pretreatment with ROS scavengers, *N*-acetylcysteine and reduced glutathione, and protein synthesis inhibitor, cycloheximide. Further, knockdown of MEK upstream regulator RAF1 and autophagosomal marker LC3, and co-treatment with JNK or ERK1/2 inhibitor resulted in the most significant attenuation of DETD-35-induced morphological and molecular or biochemical changes in cancer cells, while the inhibitory effect of DET was not influenced by MAPK inhibitor treatment. Therefore, DETD-35 exerted both ER stress-mediated paraptosis and apoptosis, which may explain its superior activity to DET against TNBC cells. Although the chemotherapeutic drug paclitaxel induced vacuole-like structures in MDA-MB-231 cells, no paraptotic cell death features were detected. This study provides a strategy for combating TNBC through sesquiterpene lactone analogs by induction of oxidative and ER stresses that cause paraptosis-like cell death.

## INTRODUCTION

Breast cancer is the most commonly diagnosed cancer in women, with 246,660 new cases diagnosed and 40,450 deaths in the United States in 2016 [1]. Although various clinical therapeutic options including primary surgery, radiotherapy, pre- or postoperative adjuvant chemotherapy, and hormonal therapy are current common treatments for breast cancer that improve survival rates, anti-cancer drug resistance, and locally advanced and metastatic breast cancer still remain clinical challenges [2, 3]. Clinically, breast cancer is divided into several different subgroups that are commonly classified based on the expression status of various receptors such as the estrogen receptor (ER), progesterone receptor (PR) and human epidermal growth factor receptor 2 (HER2), among which triple negative (ER-/PR-/HER2-) breast cancer (TNBC) reveals highly metastatic features and poor prognosis. In addition, TNBC is accompanied by higher risk of early recurrence, and shorter disease-free and overall survival compared to all other breast tumor subgroups [4, 5].

For decades, plant-derived phytocompounds alone or in combinational chemotherapy have been used as strategies for cancer prevention in various allograft or xenograft animal models. One particular phytoagent that has attracted attention recently is deoxyelephantopin (DET), a major germacranolide sesquiterpene lactone isolated from the traditional medicinal herb *Elephantopus scaber* L. or other *Elephantopus* genus plants. In Chinese medicine, *Elephantopus scaber* is used for treating hepatitis, bronchitis, nephritis, arthralgia, or stomach disease symptoms [6, 7]. In our previous studies, we observed that DET is the active compound in the medicinal plant which was found to significantly suppress mammary tumor growth and lung metastasis of TS/A (ER+) mammary cancer cells *in vivo* and *in vitro*, and restrict cancer cell activity through regulating multiple molecular mechanisms, including formation of centrosomal aggregates, deregulation of the ubiquitin-proteasome system and NF-κB activity, induction of ROS/JNK-mediated apoptosis and protein carbonylation, and restriction of cell motility by inhibiting m-calpain enzyme activity [8–10]. To further enhance and broaden the suppressive effects of DET against cancers, we created a spectrum of DET derivatives (DETDs) by semi-organic synthesis and determined the most active, which we named DETD-35 [11]. DETD-35 exhibited more potent activity than parental DET, markedly repressing tumor growth and lung metastasis in MDA-MB-231 tumor-bearing SCID mice; however, the detail molecular mechanisms underlying the activity of DET and DETD-35 were not elucidated.

Cytoplasmic vacuolation-related cell death, such as paraptosis or paraptosis-like cell death is a kind of non-apoptotic and non-autophagic programmed cell death. This type of cell death has recently been proposed as a novel therapeutic strategy which may effectively inhibit the activity of drug-resistant cancer cells that have intrinsic or adaptive resistance to apoptosis, and thus reduce the therapeutic limitations of anti-cancer agents that are primarily dedicated to induction of pro-apoptotic cell death [12–14]. However, the molecular mechanisms underlying cytoplasmic vacuolation-related paraptosis are less understood than other types of programmed cell death, particularly in the area of natural products or chemotherapeutical drug-induced effects against cancer cells [15].

In this study, we uncovered the modes of action of both parental DET and its novel derivative DETD-35 against MDA-MB-231 TNBC cells. We observed that DETD-35 significantly induced oxidative stress that subsequently facilitated ER dysfunction and paraptosis-like cell death as shown by ER-associated massive cytoplasmic vacuole formation, and ER stress-mediated apoptosis or paraptosis in cancer cells. Part of the mechanism was also observed in DET treatment. This study is the first to show that sesquiterpene lactones can promote oxidative stress-induced cytoplasmic vacuolation death mechanisms to suppress TNBC cell activity.

## RESULTS

### DET and DETD-35 inhibited cell proliferation and growth and induced cytoplasmic vacuolation formation in MDA-MB-231 cells

**Figure 1:**
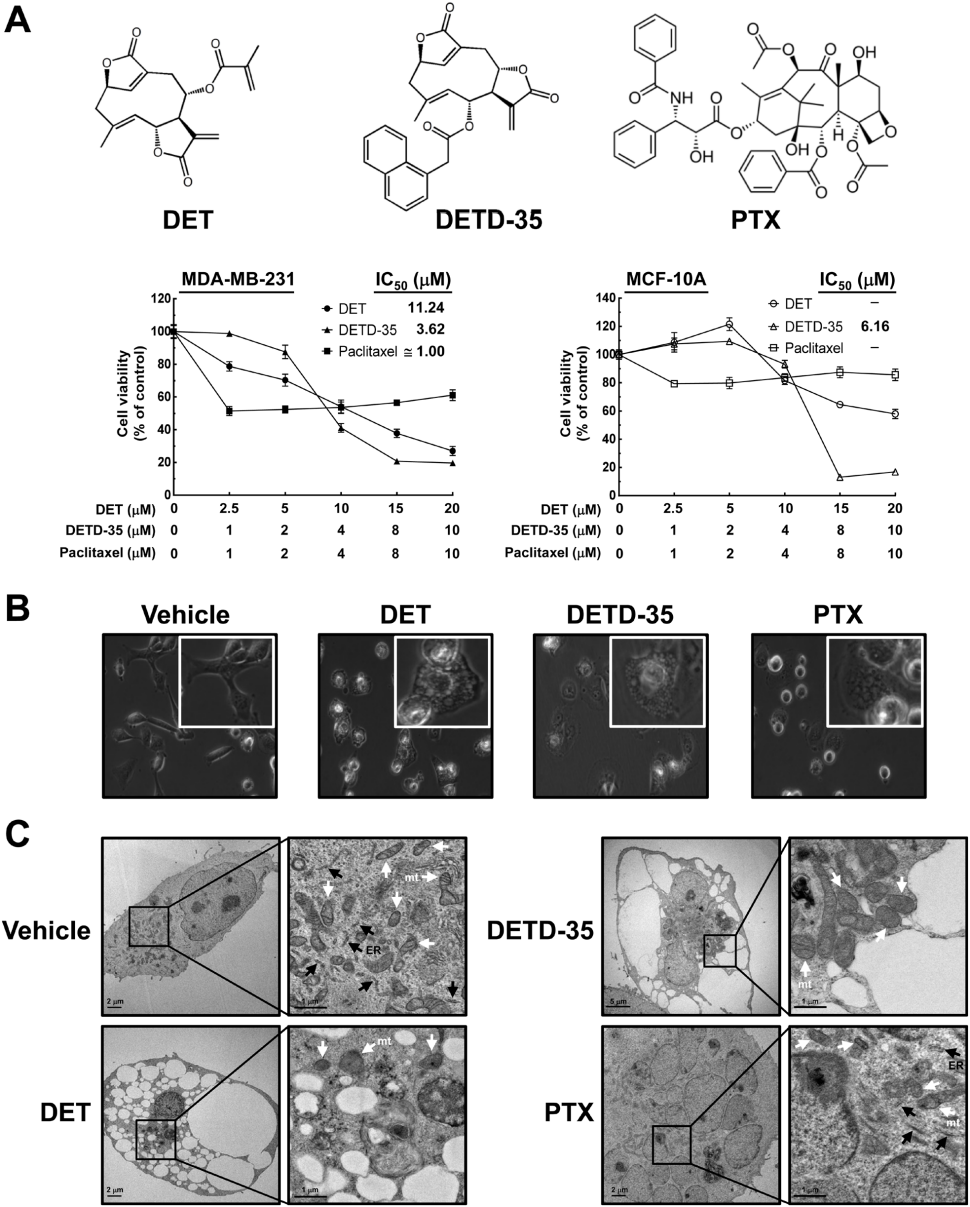
Effects of DET and DETD-35 on MDA-MB-231 cells **(A)** Chemical structure of paclitaxel (PTX), deoxyelephantopin (DET) and its derivative DETD-35; MDA-MB-231 and MCF-10A cells were treated with the indicated concentrations of DET, DETD-35, and PTX for 24 h, and then the cell viability was examined using MTT assay. **(B)** MDA-MB-231 cells were treated with vehicle (0.5% DMSO), DET (11 μM), DETD-35 (3 μM), and PTX (1 μM) for 24 h, and the morphological changes of cancer cells were examined by light microscopy (×400 magnification). **(C)** Transmission electron microscopy (TEM) imaging (×10,000 magnification) of untreated (vehicle) and treated (DET, 11 μM; DETD-35, 3 μM; PTX, 1 μM) MDA-MB-231 cells. The ER and mitochondria (mt) are indicated by black arrowheads and white arrowheads, respectively.

The effect of DET and DETD-35 on human TNBC cell proliferation and phenotype were investigated. The cytotoxicity of DET, DETD-35, and paclitaxel (PTX), a chemotherapeutic drug for TNBC used as a reference, were examined at the indicated concentrations for 24 h. DETD-35 was a more potent suppressor of MDA-MB-231 cell viability than the parental compound DET with 50% inhibition concentrations (IC_50_) determined as 3.62 and 11.24 μM, respectively. PTX reduced cell viability by about 50% at around 1 μM. Both compounds had little effect (∼20% inhibition) on proliferation of a normal human mammary epithelial cell line (MCF-10A) at concentrations around their IC_50_ values against cancer cells; however, some cytotoxicity was detected when compound concentration was increased (Figure 1A). Previously, we examined the *in vivo* effect of both compounds against MDA-MB-231 cell activity in an orthotopic tumor model using NOD/SCID mice [11]. We observed that treatment with DETD-35 (10 mg/kg/every three days, *i.p*.) or DET (20 mg/kg/every three days, *i.p*.) suppressed tumor size 40-44% compared to the tumor control group with vehicle treatment, and there was no significant difference in the body weight of test mice in any of the groups. In this study, the levels of proliferation marker Ki-67 were examined in tumor tissues and showed significant inhibition in DET- and DETD-35-treated groups compared to the tumor control group (*P* < 0.05) (Supplementary Figure 1). The *in vitro* and *in vivo* data demonstrate that DETD-35 has a more potent effect than the parental DET against triple negative breast cancer cell proliferation and growth.

Further, both DET and DETD-35 at 11 μM and 3 μM, respectively, significantly induced the formation of massive cytoplasmic vacuoles in the perinuclear region of MDA-MB-231 cells treated for 24 h, as examined by light microscopy. PTX treatment (1 μM) also generated some vacuole-like structures near the nuclear region of MDA-MB-231 cells (Figure 1B). We further examined the detailed morphology of treated TNBC cells using transmission electron microscopy (TEM). As shown in Figure 1C, after treatment for 24 h, several empty vacuoles had appeared in DET- and DETD-35-treated MDA-MB-231 cells with the plasma membrane retained intact, but with a lack of detectable cytoplasmic materials. PTX treatment induced the appearance of multiple micronuclei within cells, and generated several vacuole-like structures containing rich and dense contents; different from the observations for DET or DETD-35 treatment (Figure 1C). The multiple ribosomes embedded on the rough endoplasmic reticulum (RER) membrane, a feature of RER structures, were found in the vehicle and PTX-treated TNBC cells, but were not seen after either DET or DETD-35 treatment. Meanwhile, both DET and DETD-35 caused significant damage to the mitochondrial structures in the treated TNBC cells. A large population of swollen mitochondria was observed in DETD-35-treated cells and severe damage to mitochondria structural integrity was observed in DET-treated cells in comparison to vehicle-treated cells. PTX treatment did not cause any apparent mitochondrial damage, except obvious multi-nuclei formation. Together, these results indicate that both DET and DETD-35 treatment induced the formation of massive cytoplasmic vacuoles and damaged the integrity of ER and mitochondrial structures in human TNBC cells; and the effect seen was obviously different from the PTX effect.

### DETD-35 promotes non-autophagic cytoplasmic vacuolation death in TNBC cells

To further pinpoint the potential molecular mechanisms of DET- and DETD-35-induced cytoplasmic vacuolation in inhibition of TNBC cell activity, we first examined whether compound-stimulated cytoplasmic vacuolation is related to autophagic cell death. The accumulation of autophagic vacuoles has been reported to promote cancer cell death through deregulation of lysosomal membrane permeabilization [16]. In addition, the different stages of the autophagic process can be indicated using distinct autophagy marker proteins. We, therefore, used two different kinds of autophagy inhibitor, 3-methyladenine (3-MA), an inhibitor that inhibits autophagy by blocking pre-autophagosome formation via the inhibition of class III phosphatidylinositol-3 kinase (PI3K), and bafilomycin A1 (BAF-A1), a vacuolar-type H^+^-ATPase (V-ATPase) inhibitor that inhibits fusion between autophagosomes and lysosomes. Further, we co-treated the cancer cells with either DET or DETD-35 and measured cell viability, monitored cell morphology, and examined beclin-1 (an early stage marker of autophagy), LC3 (microtubule-associated protein 1 light chain 3, an autophagosomal marker) and p62 (a marker of autophagic flux at a later stage of autophagy) protein expressions in MDA-MB-231 cells.

**Figure 2:**
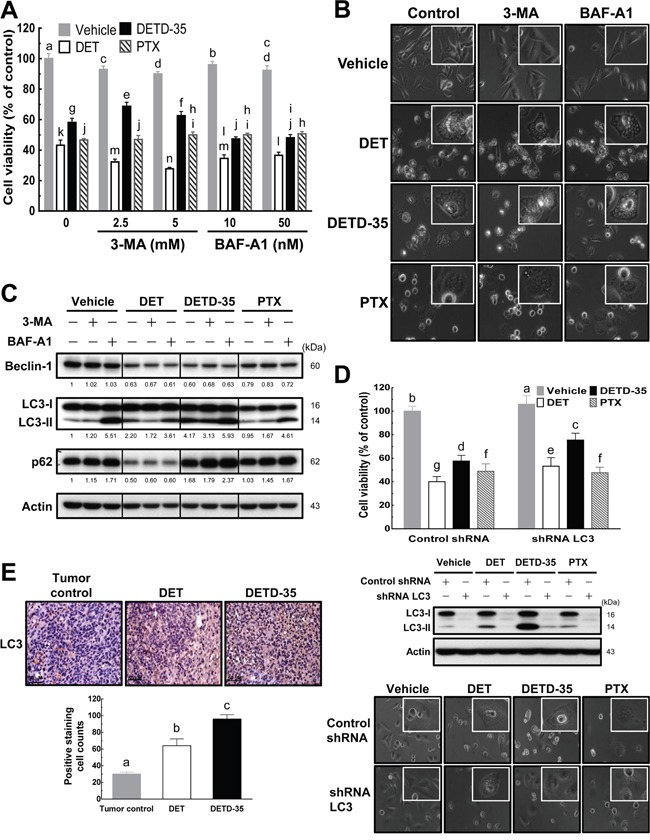
DET and DETD-35 induce nonautophagic cytoplasmic vacuolation death in TNBC cells **(A)** Autophagy inhibitors, 3-methyladenine (3-MA) or bafilomycin A1 (BAF-A1), were co-treated with either vehicle (0.5% DMSO) or compounds (DET, 11 μM; DETD-35, 3 μM; PTX, 1 μM) for 24 h. The viability of treated MDA-MB-231 cells was determined using MTT assay. Different letters represent significant differences (one-way ANOVA, *P* < 0.05). **(B)** MDA-MB-231 cells were treated with vehicle and compounds alone, or compounds with autophagy inhibitors (2.5 mM 3-MA and 10 nM BAF-A1) for 24 h, and then the morphological changes in the treated cells were observed using light microscopy (×400 magnification). **(C)** Whole cell protein extracts were prepared after the various treatments described in **(B)**, and the expression levels of three different autophagy marker proteins, beclin-1, LC3-II, and p62, were further examined by western blotting. Quantification of the corresponding protein bands was performed using Image Studio Lite software. β-actin was used as a loading control. **(D)** LC3 gene expression was knocked down using shRNA (shRNA LC3), and shLacZ was used as a control shRNA. The shRNA-transfected TNBC cells were treated with vehicle (0.5% DMSO), DET (11 μM), DETD-35 (3 μM), and PTX (1 μM) for 24 h, and then subjected to MTT assay and light microscopy to check the cell viability and morphological changes in treated TNBC cells. The transfected TNBC cell lysates were immunoblotted with LC3 antibodies. β-actin was used as a loading control. **(E)** Representative immunohistochemical images of orthotopic MDA-MB-231 tumor sections from tumor control (without treatment) and DET or DETD-35 treated NOD/SCID mice. The positive LC3 staining was visualized as a brownish color, and nuclei were stained by hematoxylin and shown in blue color. Scale bar represents 50 μm. Data are mean ± SEM, *n* = 3. Different letters represent significant differences (one-way ANOVA, *P* < 0.05).

As shown in Figure 2A, co-treatment with either 3-MA or BAF-A1 slightly affected DET (−10%) or DETD-35 (±10%) cytotoxicity against MDA-MB-231 cells, while no impact was observed in PTX-treated cells. Light microscopy showed that co-treatment with these autophagy inhibitors did not diminish the ability of DET and DETD-35 to induce cytoplasmic vacuolation in MDA-MB-231 cells (Figure 2B), suggesting that the cytoplasmic vacuolation-mediated cell death induced by both compounds in MDA-MB-231 cells may be through a non-autophagic type of cell death. Further, western blot analysis of the expression level of autophagy marker proteins in the control or treated cells revealed that, in the presence or absence of 3-MA or BAF-A1 inhibitor, DET-suppressed beclin-1 and p62 protein levels did not change, but DET-induced increased protein expression of LC3-II, an autophagic vesicle form associated marker, was slightly attenuated by co-treatment with 3-MA (Figure 2C), while co-treatment with BAF-A1 enhanced the protein level of DET-stimulated LC3-II. Neither autophagy inhibitor had an influence on DETD-35-reduced beclin-1 expression, but 3-MA slightly decreased LC3-II accumulation, and BAF-A1 enhanced LC3-II and p62 accumulation in the DETD-35-treated cells (Figure 2C). On the other hand, cancer cells treated with PTX alone decreased the beclin-1 level which was not affected when cells were co-treated with PTX plus either inhibitor, in comparison with the control. Interestingly, PTX co-treated with BAF-A1 affected the expression level of LC3-II and p62 more significantly. Overall, the data reveal that treatment with either DET or DETD-35 induced an incomplete autophagic process in cancer cells.

To further confirm the role of autophagosomal LC3-II in DET- and DETD-35-induced cytoplasmic vacuolation, we knocked down LC3 expression in MDA-MB-231 cells using small hairpin RNA (shRNA) (Figure 2D). We observed partially increased viability of treated LC3-knockdown TNBC cells along with some degree of reduction of cytoplasmic vacuoles in both DET (13%) and DETD-35 (18%) treatment due to reduced accumulation of autophagosomal LC3-II. No changes were observed in the PTX-treated cells with or without knockdown of LC3-II (Figure 2D). Next, we examined whether the autophagosome accumulation stimulated by either compound observed in the cell line also appeared in MDA-MB-231 tumor specimens collected from the xenograft mice with either DET or DETD-35 treatment from our recent animal study [11]. Immunohistochemical (IHC) analysis showed that the expression level of autophagosomal marker LC3 significantly increased in DET- and DETD-35-treated TNBC tumor specimens (Figure 2E), which perfectly matched the markedly induced autophagosomal protein accumulation in MDA-MB-231 cells *in vitro*. Taking both the *in vitro* and *in vivo* data together, we conclude that DET derivatives, especially DETD-35 induced non-autophagic cytoplasmic vacuolation-mediated cell death in MDA-MB-231 cells, which was at least in part through significant induction of autophagosome (LC3-II) accumulation.

### DET- and DETD-35-caused cytoplasmic vacuolation death is associated with endoplasmic reticulum (ER) stress and ubiquitinated protein accumulation

**Figure 3:**
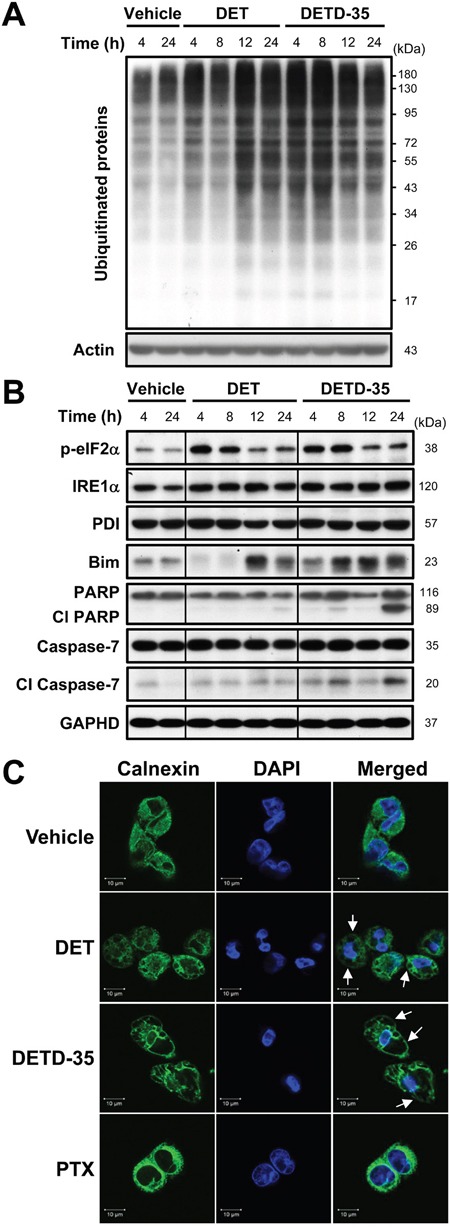
DET and DETD-35 significantly induce ubiquitinated protein accumulation, and ER stress and dilation in MDA-MB-231 cells **(A)** Immunoblotting of ubiquitinated proteins in MDA-MB-231 cells treated with vehicle (0.5% DMSO), DET (11 μM), or DETD-35 (3 μM) for 4, 8, 12, and 24 h. The expression level of β-actin was used as a loading control. **(B)** The protein level of ER stress-related proteins, p-eIF2α, IRE1α, PDI, and Bim, and apoptosis-related proteins, the cleaved form of PARP and caspase-7, in vehicle or compound treated cells were determined by western blotting. GAPDH was used as a loading control. **(C)** Immunofluorescence analysis of MDA-MB-231 cells treated with vehicle (0.5% DMSO), DET (11 μM), DETD-35 (3 μM), or PTX (1 μM) for 24 h. The treated cells were fixed using 100% ice-cold methanol, and stained with calnexin, an ER-membrane bound protein (green), and DAPI (blue) to visualize ER structures and nuclei. White arrows indicate DET- and DETD-35-induced cytoplasmic vacuoles.

According to the light microscopy and TEM analysis results, DET and DETD-35 treatment was able to disrupt the ER structure in MDA-MB-231 cells, possibly as a result of ER stress. Previously, ER stress and protein ubiquitination have been associated with cytoplasmic vacuolation-mediated cell death [15]. We thus further examined whether both DET- and DETD-35-induced cytoplasmic vacuolation accompanies accumulation of ubiquitinated proteins and ER stress-related protein increase in MDA-MB-231 cells. The western blotting result revealed that both compounds markedly promoted the accumulation of ubiquitinated proteins in treated MDA-MB-231 cells relative to the control cells. DETD-35 treatment stimulated a relatively greater degree of ubiquitinated protein accumulation at short treatment durations (4 and 8 h); whereas, DET treatment induced more at 12 and 24 h (Figure 3A). We next checked for ER damage and/or misfolded or unfolded protein accumulation that could trigger ER stress and the related signaling pathways such as unfolded protein response (UPR) signaling. We observed that DET and DETD-35 significantly up-regulated ER stress-related proteins, including the phosphorylated form of eukaryotic initiation factor-2α (eIF2α), a well-known substrate of protein kinase RNA (PKR)-like ER kinase (PERK), at 4-8 h treatment and inositol-requiring protein-1α (IRE1α), one of the major UPR signaling molecules (Figure 3B). Meanwhile, both DET and DETD-35 treatments were also able to promote the expression of Bim, a pro-apoptotic BH3-only group of Bcl-2 related proteins that plays an essential role in ER stress-mediated apoptosis. Interestingly, the protein level of protein disulfide isomerase (PDI), an ER-associated enzyme, was not altered by treatment with either compound. Next, we examined the expression of programmed cell death hallmarks, poly ADP-ribose polymerase (PARP) and caspase-7. We observed that, within 24 h, DETD-35 treatment caused more apoptotic cell death than DET treatment in MDA-MB-231 cells. Besides inducing ER stress, overloading of misfolded proteins in the ER lumen of cells may also cause ER dilation which is positively associated with cytoplasmic vacuolation. Therefore, immunofluorescence staining of the ER-membrane bound protein, calnexin, was conducted in control and treated cancer cells. As shown in the confocal fluorescence images in Figure 3C, there was positive and evenly distributed calnexin staining (green) in the cytoplasm of vehicle control cells. Strikingly, in DET- and DETD-35-treated cells, the positive calnexin staining took place at the membrane surrounding the enlarged vacuoles in the cytosol, suggesting that both DET- and DETD-35-induced cytoplasmic vacuoles originated from the ER in MDA-MB-231 cells. Such ER dilation and derived vacuoles were not observed in PTX-treated cells. Together, these findings indicate that both DET- and DETD-35-induced cytoplasmic vacuolation and cell death were associated with de-regulation of ubiquitin proteasome proteolysis and ER function and the associated stress signaling.

### DET- and DETD-35-induced cytoplasmic vacuolation require protein synthesis

**Figure 4:**
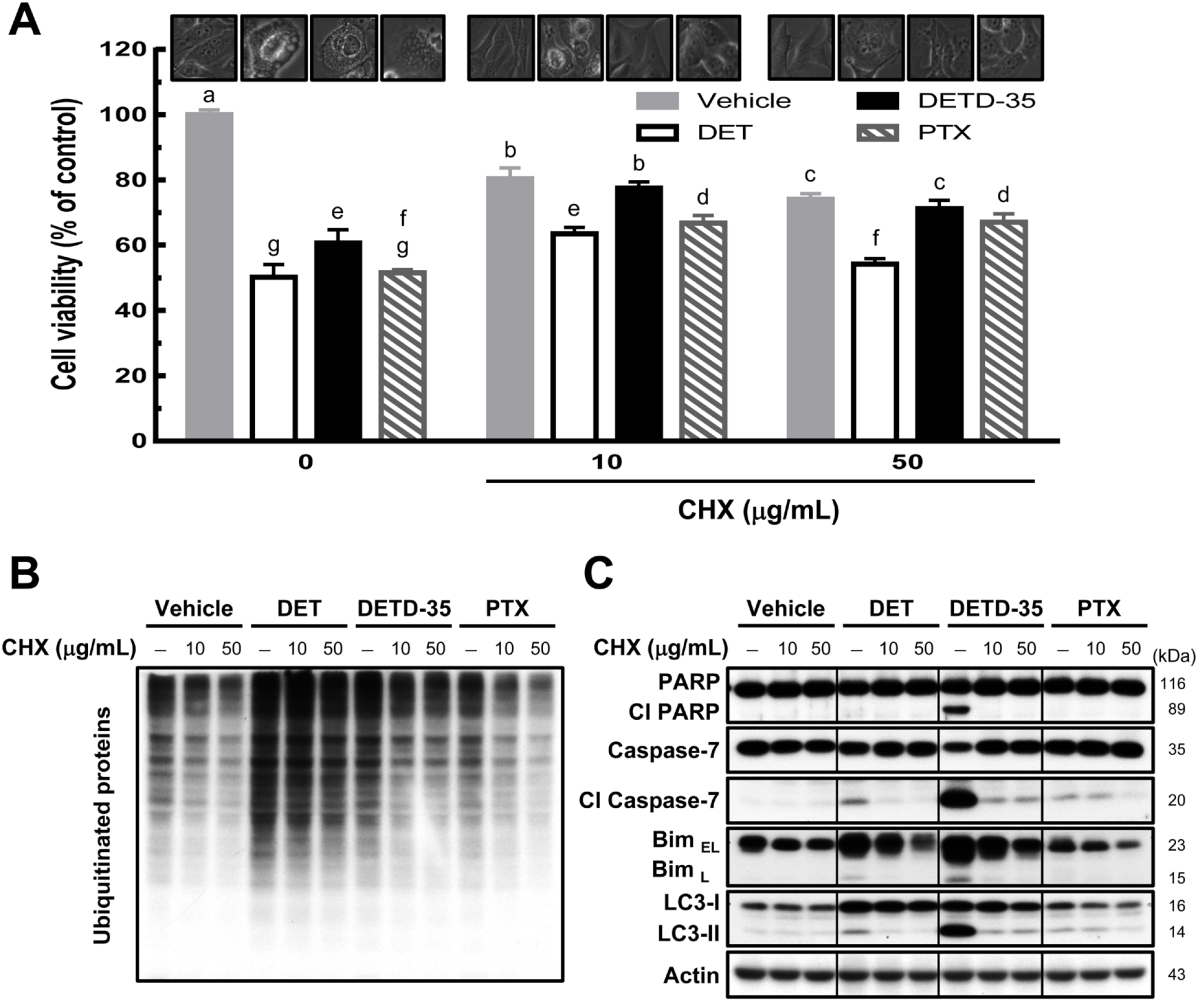
Pretreatment of protein synthesis inhibitor prevents DET- and DETD-35-triggered cytoplasmic vacuolation death in TNBC cells **(A)** MDA-MB-231 cells were pretreated with protein synthesis inhibitor, cycloheximide (CHX 10 or 50 μg/mL), for 1 h, and subsequently treated with vehicle (0.5% DMSO), DET (11 μM), DETD-35 (3 μM), and PTX (1 μM) for 24 h. Cell viability was determined using MTT assay. Different letters represent significant differences (one-way ANOVA, *P* < 0.05). Phase-contrast microscopic images of the compound and/or CHX effect on vacuole formation in TNBC cells. Immunoblotting of ubiquitinated protein profile and levels **(B)** and the levels of apoptosis-related proteins (cleaved PARP and cleaved caspase-7), ER stress-associated protein (Bim), and autophagosomal marker protein LC3-II **(C)** in treated TNBC cells. β-actin was used as a loading control.

As we observed ER dilation in MDA-MB-231 cells treated with DET derivatives, it was worth investigating whether the protein synthesis machinery is associated with the cytoplasmic vacuolation-related cell death caused by DET and DETD-35. Protein synthesis inhibitor, cycloheximide (CHX) was used to pretreat MDA-MB-231 cells followed with or without compound treatment. Cell viability, cytoplasmic vacuolation and ubiquitinated protein levels in the cancer cells were examined. Pretreatment with CHX (10 and 50 μg/mL) in vehicle control cells showed some inhibition (< 24%) of MDA-MB-231 cell proliferation compared to the vehicle control in the absence of CHX (Figure 4A). CHX pretreatment restored DET-, DETD-35-, and PTX-inhibited cell viability by around 14-17% compared to compound or PTX treatment alone, while CHX at 10 μg/mL prevented the formation of vacuole structures most significantly in DETD-35-treated cells. A more potent effect was detected for 50 μg/mL CHX pretreatment in DET-treated cells (Figure 4A). Furthermore, pretreatment with a low concentration (10 μg/mL) of CHX markedly reduced DETD-35-induced ubiquitinated protein accumulation and the expression levels of Bim, autophagosomal marker LC3-II and cleaved forms of PARP and caspase-7 in TNBC cells compared to DET or PTX treatment (Figure 4B and 4C). These results demonstrated that treatment with protein synthesis inhibitor CHX can partially neutralize the ability of DET, and DETD-35 to repress TNBC cell activity and programmed cell death.

### Role of MAP kinases in DET- and DETD-35-induced cytoplasmic vacuolation death in TNCB cells

**Figure 5:**
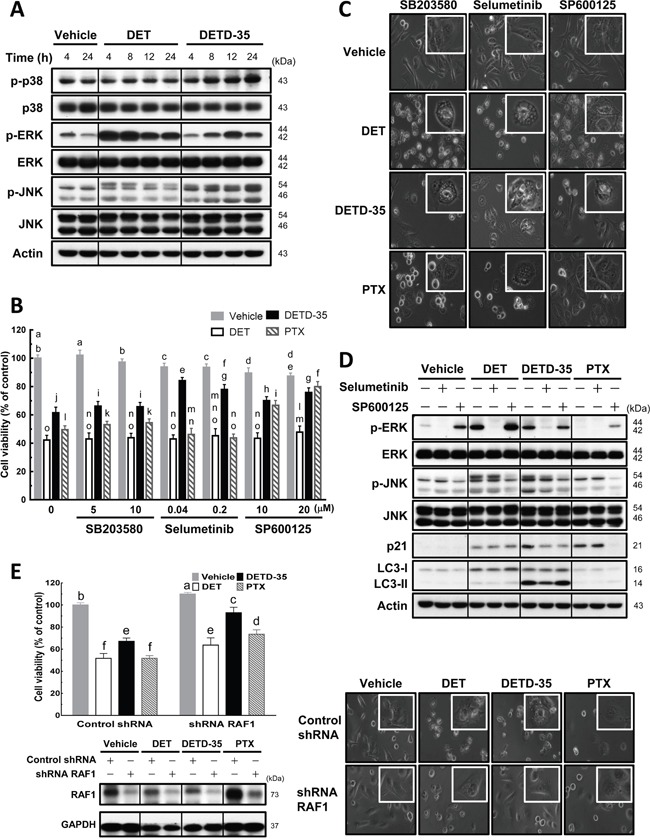
Effects of MAPK inhibitors on DET, DETD-35, and PTX-induced cytoplasmic vacuole formation in TNBC cells **(A)** MDA-MB-231 cells were lysed after treatment with vehicle (0.5% DMSO), DET (11 μM), and DETD-35 (3 μM) for the indicated time periods (4, 8, 12, and 24 h), and then subjected to western blotting to determine the expression levels of original and phosphorylated forms of p38, ERK, and JNK. β-actin was used as a loading control. **(B)** The indicated concentrations of MAPK inhibitors, SB203580 for p38; selumetinib for ERK1/2; SP600125 for JNK, were co-treated with either vehicle (0.5% DMSO), DET (11 μM), DETD-35 (3 μM), or PTX (1 μM) in MDA-MB-231 cells for 24 h. Cell viability was assessed using MTT assay. Different letters represent significant differences (one-way ANOVA, *P* < 0.05). **(C)** Phase-contrast microscope images show the influence of MAPK inhibitor (SB203580, 5 μM; selumetinib, 0.04 μM; SP600125, 20 μM) co-treatment with compound on vacuole formation. **(D)** Immunoblotting of ERK, p-ERK, JNK, p-JNK, JNK substrate p21^WAF1/Cip1^, and autophagosomal marker protein LC3-II in TNBC cells treated with vehicle and compound alone, or compound plus either selumetinib (0.04 μM) or SP600125 (20 μM) for 24 h. β-actin was used as a loading control. **(E)** Knockdown of RAF1 gene expression using shRNA (shRNA RAF1), and shLacZ was used as a control shRNA in the experiment. The shRNA-transfected TNBC cells were treated with vehicle (0.5% DMSO), DET (11 μM), DETD-35 (3 μM), and PTX (1 μM) for 24 h, and then subjected to MTT assay and light microscopy to evaluate the cell viability and morphological changes in treated TNBC cells. Transfected TNBC cell lysates were immunoblotted to validate the knockdown efficiency of shRNA RAF1 in vehicle or compound-treated cells. GAPDH was used as a loading control.

Activation of mitogen-activated protein kinases (MAPKs) has often been described to be involved in paraptosis/paraptosis-like cell death and/or cytoplasmic vacuolation-mediated cell death in cancer cells [12, 17]. Therefore, we examined the activation status of MAPKs during DET and DETD-35 treatment at 4 to 24 h, and found that both compounds can significantly enhance both phosphorylation levels of extracellular signal-regulated kinase (ERK) and c-Jun N-terminal kinase (JNK). In comparison, DET had a more significant effect on phospho-ERK at shorter treatment times (4-8 h) while DETD-35 promoted phospho-JNK expression time-dependently from 4 to 24 h treatment; moreover, DETD-35 also increased phospho-p38 level; this increase was not observed in DET-treated cells (Figure 5A). Based on the western blot data, we decided to employ various MAP kinase inhibitors, i.e., SB203580 (p38 MAP kinase inhibitor), selumetinib (ERK1/2 or MEK1 inhibitor) and SP600125 (JNK inhibitor), to investigate whether specific MAP kinase activity is associated with the compound effects on anti-TNBC cell proliferation and/or the induction of cancer cell cytoplasmic vacuolation and cell death. First, we observed that the three MAPK inhibitors did not affect the DET cytotoxicity against MDA-MB-231 cells, but ERK1/2 inhibitor and JNK inhibitor co-treatment attenuated approximately 23% and 14%, respectively of DETD-35 cytotoxic activity, interestingly JNK inhibitor reversed ∼30% of PTX cytotoxicity (Figure 5B). On the other hand, co-treatment with either ERK1/2 or JNK inhibitor reduced DETD-35-induced cytoplasmic vacuole formation in TNBC cells (Figure 5C). This result is in good agreement with the reversal effect of the inhibitors on the compound cytotoxicity (Figure 5B). Furthermore, a reversal effect of JNK inhibitor on PTX cytotoxicity and PTX-induced vacuole-like structures in MDA-MB-231 cells was also observed (Figure 5B and 5C). Although we observed the overphosphorylation of ERK protein with DET treatment, we could not detect the ERK1/2 inhibitor effect on DET cytotoxicity and DET-induced cytoplasmic vacuolation. Meanwhile, the p38 MAP kinase inhibitor did not significantly affect the cytotoxicity of DET, DETD-35, and PTX and their induced vacuole formation and cell death in the treated TNBC cells. Therefore, we employed the ERK1/2 and JNK inhibitor, selumetinib and SP600125, respectively, to further confirm whether both MAPK inhibitors could abolish both compound-stimulated ERK and JNK activation. Indeed, western blot analysis revealed that the inhibition of phospho-MAPKs with selumetinib and SP600125 blocked DET/DETD-35-stimulated overexpression of phospho-ERK and phospho-JNK in MDA-MB-231 cells; meanwhile, both inhibitors significantly decreased DETD-35-induced expression of JNK substrate p21^WAF1/Cip1^ and only JNK inhibitor affected PTX-induced p21^WAF1/Cip1^ level, whereas no effect was observed in DET treatment. Interestingly, LC3-II protein level was only attenuated by ERK1/2 inhibitor in DETD-35-treated cells (Figure 5D), suggesting the overexpression of phospho-ERK during DETD-35 treatment may partially participate in the mechanism of DETD-35-induced autophagosome accumulation in treated cells. These data suggest that ERK and JNK are involved in the DETD-35- and DET-mediated cytoplasmic vacuolation in TNBC cells to a different degree.

We further knocked down v-raf-1 murine leukemia viral oncogene homolog 1 (RAF1), an upstream regulator gene of the MEK signaling pathway, using shRNA to see whether the compound cytotoxicity or induction of vacuole formation in cancer cell cytoplasm was affected. The results, shown in Figure 5E, reveal an attenuation of DET (12%), DETD-35 (26%), and PTX (22%) cytotoxicity by knockdown of RAF1 in the cancer cells. A more significant reversion of the vacuoles formed in the cytoplasm was seen with DETD-35 treatment than with DET or PTX treatment (Figure 5E). The data indicate that RAF1-mediated MEK signaling networks are likely involved in DETD-35-induced cytoplasmic vacuolation death in human TNCB cells.

### DET- or DETD-35-induced ROS mediates cytoplasmic vacuolation-associated cell death

**Figure 6:**
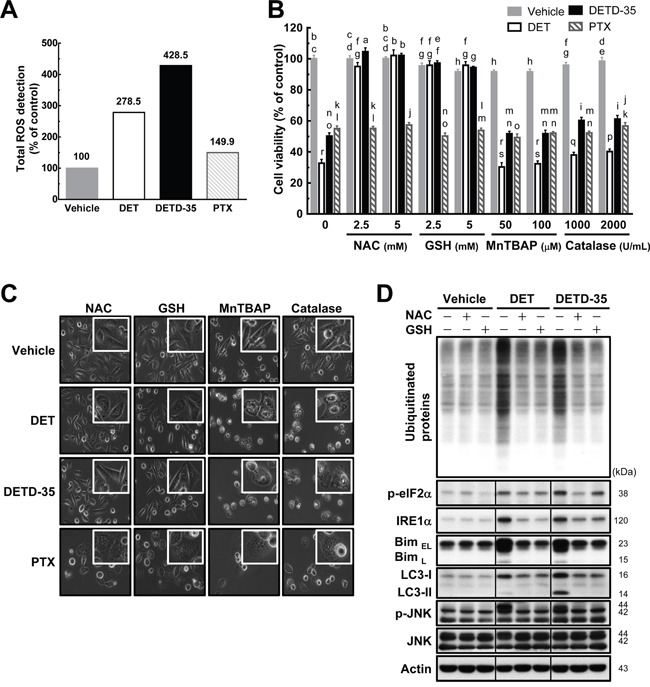
Pretreatment with either NAC or GSH prevents DET and DETD-35 induced ROS production and cytoplasmic vacuolation-mediated cell death **(A)** MDA-MB-231 cells were treated with vehicle (0.5% DMSO), DET (11 μM), DETD-35 (3 μM), and PTX (1 μM) for 1 h. The detection of ROS production in the treated cells is described in the Materials and Methods section. **(B)** MDA-MB-231 cells were treated with the indicated concentrations of ROS scavengers, *N*-acetylcysteine (NAC), reduced glutathione (GSH), Mn(III)tetrakis (4-benzoic acid) porphyrin chloride (MnTBAP), and catalase-polyethylene glycol (catalase-PEG) for 1 h, and then treated with the vehicle and compounds for 24 h. Cell viability was assessed using MTT assay. Different letters represent significant differences (one-way ANOVA, *P* < 0.05). **(C)** Phase-contrast microscopic images show the influence of ROS scavenger (NAC, 5 mM; GSH, 5 mM; MnTBAP, 50 μM; catalase, 1000 U/mL) pre-treatment alongside compound treatment on cytoplasmic vacuole formation. **(D)** Immunoblotting of ubiquitinated proteins, ER stress-associated proteins (phospho-eIF2α, IRE1α, and Bim), LC3-II, JNK, and p-JNK in the TNBC cells pre-treated with NAC (5 mM) and GSH (5 mM) for 1 h, and then treated with vehicle or compounds for 24 h. β-actin was used as a loading control.

We observed previously that DET or DETD-35 treatment could induce the production of reactive oxygen species (ROS) in mouse TS/A mammary cancer cells [9] or human BRAF mutant A375 melanoma cells [18]. The cytotoxic effect of both compounds was attributed to this ROS production. We thus further evaluated whether DET and DETD-35 can increase ROS level in the human TNBC MDA-MB-231 cells and cause the activation of ER stress-related pathways and cytoplasmic vacuolation-associated cell death in the cancer cells. As shown in Figure 6A, treatment with DET or DETD-35 significantly triggered ROS production in MDA-MB-231 cells. ROS production was also found in PTX-treated MDA-MB-231 cells, but at a far lower level than with DET or DETD-35 treatment. Next, the cancer cells were pretreated with various ROS scavengers such as *N*-acetylcysteine (NAC), thiol antioxidant (reduced glutathione, GSH), manganese superoxide dismutase (SOD) mimetic [Mn(III) tetrakis (4-benzoic acid) porphyrin chloride, MnTBAP], and H_2_O_2_ scavenger (catalase-polyethylene glycol) before DET, DETD-35, or PTX treatment. NAC and thiol antioxidant GSH pretreatment almost completely abrogated the DET and DETD-35 cytotoxicity and the cytoplasmic vacuoles they induced; whereas, pretreatment with MnTBAP and H_2_O_2_ scavenger catalase did not show any effect (Figure 6B and 6C). Of note, pretreatment with the four different ROS scavengers did not reverse the formation of vacuole-like structures or proliferation activity in PTX-treated TNBC cells. On the other hand, the accumulation of ubiquitinated proteins, ER stress-related proteins (IRE1α and Bim), autophagosomal marker LC3-II, and phospho-JNK in the cells under DET and DETD-35 treatment were also significantly counteracted by pretreatment with NAC or GSH (Figure 6D), indicating that DET and DETD-35 treatment can facilitate a specific type of ROS production, apart from MnSOD-produced ROS and H_2_O_2_. This ROS production is undoubtedly a crucial upstream initiator of the suppressive effects of DET and DETD-35 against TNBC cell activity. Collectively, these results show that cytoplasmic vacuolation-mediated cell death upon DET and DETD-35 treatment in TNBC cells is partly through the ROS-mediated ER stress signaling pathways.

## DISCUSSION

Anti-cancer drug-induced apoptosis is one of the mechanisms most commonly utilized in cancer therapy; however, cancer cells may develop different adaptive or innate mechanisms, such as modification and mutation of drug targets, alteration of drug metabolism and transport, enhancement of DNA repair, and dyregulation of apoptotic proteins, to promote the formation of apoptosis-resistant cancer cells resulting in severely limited anti-cancer drug efficacy [19]. Recently, targeting several forms of non-apoptotic programmed cell death (PCD), such as autophagy, paraptosis, necroptosis, pyroptosis, and cytoplasmic vacuolation-mediated cell death have been demonstrated to be effective [12, 20, 21] and have been considered as new strategies for preventing or combating malignant cancer.

Various natural products, such as curcumin and paclitaxel, have been reported to function as anti-cancer agents by inducing paraptotic PCD in cancer cells through inducing extensive cytoplasmic vacuolation beginning from progressive organelle ER and/or mitochondria dilation [12]. In this study, we observed for the first time that plant-derived sesquiterpene lactone DET and its derivative could significantly induce ER dilation and cytoplasmic vacuole formation in the triple negative breast cancer cell line MDA-MB-231. Regulation of autophagosomal marker LC3 has been found to be involved in cytoplasmic vacuolation-associated death in cancer cells [13, 22]. Our data show that DET or DETD-35 stimulated considerable accumulation of LC3-II protein in MDA-MB-231 cells as well as in the MDA-MB-231 tumor specimens collected from xenograft mice (Figure 2). Further, knockdown of LC3 expression in MDA-MB-231 cells with LC3 shRNA concurrently reduced the cytotoxicity and cytoplasmic vacuole formation caused by DET or DETD-35 in TNBC cells. Meanwhile, autophagy inhibitor BAF-A1 co-treatment also increased the anti-proliferative effect of both compounds. These results indicate that autophagosome accumulation-induced cell death plays a role in the DET/DETD-35 effect against triple negative breast cancer cells.

Autophagy is an evolutionarily conserved process in eukaryotes which responds to varied environmental stimuli such as oxidative stress, ER stress, hypoxia, and xenobiotic treatment. It participates in various basic cellular biological processes and pathologies, such as aging, differentiation, immunity, tumorigenesis, and cell death [23]. A complete autophagic process is made up of a series of sequential stages, including initiation of phagophore formation, autophagosome elongation and formation, and fusion of autophagosomes with lysosomes to form autophagolysosomes [24]. It was reported previously that knockdown of beclin-1, a protein representing the early stage of autophagy, using short interfering RNA (siRNA) can enhance ER stress-mediated paraptosis by potentiating the expression of ER stress markers CHOP and Bip in germacrane-type sesquiterpenoid 8-*p*-hdroxybenzoyl tovarol-treated HeLa cells [25]. In this study, both promoting LC3-II protein expression for autophagosome formation, and de-regulating the beclin-1 protein by the germacranolide sesquiterpene lactone DET and its derivative in TNBC cells are likely correlated to ER stress-mediated paraptosis induced by both compounds (Figure 3).

p62, a marker of autophagic flux, was significantly decreased in DET-treated cells. Besides acting as an autophagic adaptor, p62 has also been demonstrated to be a multifunctional signaling adaptor of activation of NF-κB-mediated inflammatory responses, NRF2-activated antioxidant defense, and mTORC1-dependent nutrient sensing [26]. Our previous study illustrated the novel anti-inflammatory activity of DET by deregulating NF-κB activity through competing for the hydrogen bonding of the *cis*-acting DNA element to its p65 subunit [27] that might also be referred to as the deregulation of p62 in TNBC cells. 3-MA can prevent the formation of the pre-autophagosomal structure at the early stage of autophagy, and BAF-A1 can disrupt the autophagosomal-lysosomal fusion at the late stage of autophagy. In this study, we observed that co-treatment with 3-MA can indeed attenuate the autophagosomal LC3-II expression induced by both compounds. On the other hand, inhibitor BAF-A1 acting at the later stage after autophagosome formation could increase the accumulation of autophagosomal LC3-II protein in compound-treated cells, but had no effect on the expression of beclin-1 and p62 in DET-treated TNBC cells, and p62 was increased in DETD-35 treated cells. These results demonstrate that both compounds augment autophagosome accumulation to potentiate an incomplete autophagy event in TNBC cells resulting in non-autophagic, cytoplasmic vacuolation-mediated cell death.

Cytoplasmic vacuolation-mediated cell death, paraptosis, or paraptosis-like cell death by drug or natural product treatments, are often accompanied by ER stress and ubiquitinated protein accumulation, which have a cooperative reciprocal effect in repressing cancer cell activity [12, 15]. According to the light microscopy data and TEM analysis carried out in this study, both DET and DETD-35 treatment not only resulted in morphological changes by inducing cancer cell swelling but also damaged the integrity of the ER causing accumulation of ubiquitinated proteins and the upregulated the expression of ER stress-related proteins in treated cells. These data suggest DET/DETD-35 disrupted the ubiquitin-proteasome system thus facilitating ER stress and caused accumulation of unfolded and misfolded proteins within the ER lumen that may result in ER stress-associated apoptotic or non-apoptotic programmed cell death in MDA-MB-231 cells. The extensive cytoplasmic vacuole formation, similar to that characteristic of paraptotic PCD, triggered by both compounds originated from the dilated ER structures. Protein synthesis inhibitor, cycloheximide (CHX) is known to play a role in preventing the dilation of cellular organelles, like the ER and/or mitochondria. Dilation of organelles is regarded as one of morphological features of paraptosis/paraptosis-like cell death and cytoplasmic vacuolation-mediated cell death [12, 28]. We observed that CHX markedly diminished both DET- and DETD-35-induced cytoplasmic vacuolation, ubiquitinated protein accumulation, and autophagosomal LC3-II and cell-death marker expression, phenomena that perfectly match the biochemical and morphological characteristics of paraptosis-like cell death [12].

Recently, we have demonstrated that DET- and/or DETD-35-induced reactive oxygen species (ROS) production in mouse mammary cancer cells TS/A, or human BRAF mutant A375 melanoma cells [9, 18] resulting in cancer cell apoptosis. Therefore, we attempted to explore whether DET-/DETD-35-induced oxidative stress can confer paraptosis PCD in TNBC cells. Our data showed that DET, DETD-35, and PTX significantly promoted the generation of ROS, which could be reversed by pretreatment with antioxidant NAC or thiol antioxidant GSH, suggesting that the oxidative stress triggered by both compounds may be through covalent modification of sulfhydryl groups of cellular proteins, subsequently leading to the disruption of intracellular redox homeostasis; however, the role of thiol redox homeostasis in DET- and DETD-35-treated TNBC cells is still unclear and may warrant future investigation. Further, NAC and GSH can completely reverse DET- and DETD-35-induced cytotoxicity, cytoplasmic vacuolation, and the expression of related protein markers involved in ER stress or autophagosome accumulation. Interestingly, NAC or GSH pretreatment also completely abrogated the up-regulated expression of phosphorylated form of JNK in TNBC cells treated with either compound, but DET co-treated with JNK inhibitor SP600125 had little or no effect on the DET cytotoxicity. In addition, DET up-regulated JNK substrate p21^WAF1/Cip1^. Previously we observed that co-treatment of DET with JNK inhibitor SP600125 notably reduced DET induced p21^WAF1/Cip1^ and cleaved forms of PARP and caspase-3 in TS/A cells [8], indicating that DET acts differentially on the JNK activation and JNK-mediated signaling in different breast cancer cell types. On the other hand, insulin-like growth factor I receptor (IGFIR)-induced paraptosis was reported to be positively associated with the activation of MEK-2 and JNK [17]. Interestingly, co-treatment with JNK inhibitor SP600125 obviously attenuated the cytotoxic effect of DETD-35 and PTX in TNBC cells and its associated vacuole structure formation and p21^WAF1/Cip1^ expression. Moreover, similar inhibitory bioactivity was seen when DETD-35 was used together with ERK1/2 inhibitor selumetinib or with knockdown of tumor suppressor gene *RAF1*, a serine/threonine-protein kinase, which not only diminished the cytoplasmic vacuole formation but subsequently restored the viability of treated TNBC cells. Furthermore, ERK1/2 inhibitor co-treatment obviously reduced the up-regulated expression of autophagosomal marker LC3-II, demonstrating that the activation of MAPKs and the related signaling networks may be one of regulators that mediate DETD-35-stimulated paraptosis-like cell death. It is not clear at this stage how RAF1 and its related signaling pathways are involved in the DET/DETD-35 effect against TNBC cells.

**Figure 7:**
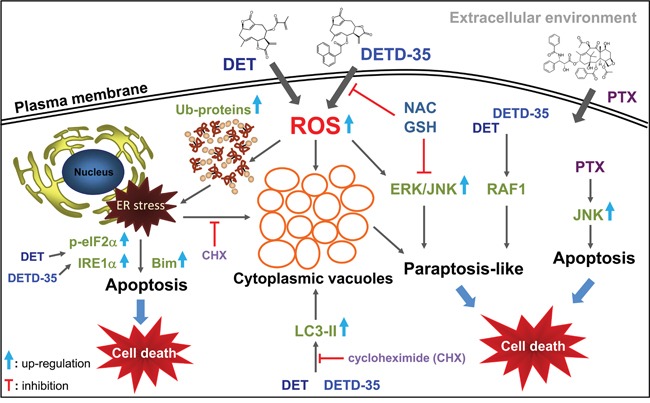
A summary of the proposed mechanisms of action of DET and DETD-35 against TNBC cells by induction of oxidative stress-mediated paraptosis-like cell death

PTX, the front-line chemotherapeutic drug for breast cancers, was used as a reference drug in this study. PTX caused vacuole-like particles or structures to occupy the nuclear space of MDA-MB-231 cells, an effect that could be reversed by CHX pre-treatment. In addition, no ER-dilation was detected in PTX-treated cells. Moreover, only a slight decrease in beclin-1 protein expression and no effect on the LC3-II and p62 proteins was observed with PTX treatment. In addition, neither the autophagic inhibitors nor ERK/MEK-1 affected the cytotoxicity of PTX in treated TNBC cells, while JNK inhibitor and CHX were able to reverse the cell viability. These findings suggest the molecular mechanism of PTX on TNBC cells is quite different from that of the DET derivatives. However, it has been reported that high concentrations of PTX (such as 70 μM) could induce paraptosis-like cell death by inducing cytoplasmic vacuolization derived from ER in ASTC-a-1 lung cancer cells [29] or in A549 tumor-xenograft mice *in vivo* [30]. It is worth mentioning, that we observed in our previous study that DET could induce ER stress and evoke ubiquitinated protein accumulation in TS/A ER(+) mammary cancer cells, but there was no ER dilation, cytoplasmic vacuoles formed, or typical paraptotic programmed cell death detected in the treated cancer cells. Instead, typical and significant ER stress-mediated apoptosis and signaling cascades were elicited [8–10]. It is very interesting that in the same way that different actions of PTX were found in lung cancer cells [29, 30] and the TNBC cells observed in this study; the same DET compound induced common apoptosis in both ER(+) TS/A cells and triple negative MDA-MB-231 cells, but only triggered distinct cytoplasmic vacuolation-associated cell death in TNBC cells. A summary of the proposed mechanisms of action of DET and DETD-35 against MDA-MB-231 cells is shown in Figure 7. This study provides a new perspective on oxidative stress-induced cytoplasmic vacuolation or paraptosis-mediated programmed cell death by a sesquiterpene lactone derivative that may constitute a promising therapeutic strategy for the treatment of apoptosis-resistant cancer or not-targetable triple negative breast cancer.

## MATERIALS AND METHODS

### Chemicals and antibodies

3-(4,5-Dimethylthiazol-2-yl)-2,5-diphenyltetrazolium bromide (MTT), dimethyl sulfoxide (DMSO), paclitaxel (PTX), bafilomycin A1 (BAF-A1), *N*-acetyl-L-cysteine (NAC), cycloheximide (CHX), catalase-polyethylene glycol (PEG), 4,6-diamidino-2-phenylindole (DAPI), and reduced glutathione (GSH) were purchased from Sigma-Aldrich. 3-Methyladenine (3-MA) and Mn(III) tetrakis (4-benzoic acid) porphyrin chloride (MnTBAP) were purchased from Merck Millipore. ERK/MEK1 inhibitor (selumetinib) was purchased from Selleck Chemicals. DMEM, FBS, and the antibiotics mixture (penicillin-streptomycin) were purchased from Invitrogen. Primary antibodies against β-actin (Chemicon, Millipore), p62 (BD Biosciences), LC3A/B and Ki-67 (Abcam), LC3B (Arigo Biolaboratories.), Raf1 (GeneTex), PDI, caspase-7, Bim, phospho-eIF2α, phospho-ERK1/2, phospho-SAPK/JNK, phospho-p38 MAPK (Cell Signaling Technology) were used. All other antibodies were obtained from Santa Cruz Biotechnology.

### Preparation of DET and its derivative DETD-35

The isolation and identification of DET from the *Elephantopus scaber* L. plant followed the protocol described by Huang et al. [8]. The synthesis of DETD-35 followed the method reported by Nakagawa-Goto et al. [11].

### Cell culture

Normal human mammary epithelial cells, MCF-10A, and triple negative human breast cancer cells, MDA-MB-231, were grown in the manufacturers’ suggested medium supplemented with 10% fetal bovine serum (FBS), 100 units/mL penicillin, and 1 mM sodium pyruvate (Gibco), and incubated in a humidified 5% CO_2_ incubator at 37°C.

### Cell viability assay

Normal and breast cancer cells (5 × 10^3^ cells/well) were seeded in 96-well culture plates and incubated overnight to allow cell adhesion. The cells were treated with vehicle (0.5% DMSO), DET, DETD-35 or PTX at the indicated concentrations for 24 h. Cell growth was determined by using 3-(4,5-dimethylthiazol-2-yl)-2,5-diphenyl tetrazolium bromide (MTT)-based colorimetric assay as previously reported [31].

### Animals

Female NOD/SCID mice (NOD.CB17-Prkdcscid/IcrCrlBltw) were supplied by the Laboratory Animal Core Facility (BioLASCO, Taiwan) and given a standard laboratory diet and distilled H_2_O *ad libitum* and kept on a 12 h light/dark cycle at 22 ± 2°C. All experimental protocols (No: 11-02-137) were approved by the Institutional Animal Care and Utilization Committee (IACUC), Academia Sinica, Taiwan, R.O.C.

### Inhibition of triple negative tumor growth in NOD/SCID mice

Triple negative mammary tumor response to DET and DETD-35 was studied using MDA-MB-231 cancer cells bearing NOD/SCID mice (6 weeks old). The mammary fat pad regions of mice were injected with 5 × 10^6^ MDA-MB-231 cells in 100 μL (9.1 mg/mL) Matrigel (Basement Membrane Matrix, Phenol Red-free) on day 0. Tumors were allowed to grow in the mice for 7 days, and then the animals were randomly assigned to three groups (*n* = 5 per group): sham, tumor control (vehicle treated), 20 mg/kg DET, and 10 mg/kg DETD-35 treatment group. DET and DETD-35 were intraperitoneally (*i.p*.) injected every three days. Mouse body weights were recorded every three days. The test mice were sacrificed by cervical dislocation at day 69, and the tumors were collected and measured for the volume (*V*) using calipers and calculated by the formula *V* = (L × W^2^)/2, where L is the length and W is the width of tumor. H&E staining and immunohistochemistry analysis of tumor tissues was carried out according to the method described by Feng et al. [18]. The sections were stained with rabbit antibody against Ki-67 or LC3A/B (Abcam) for IHC analysis. The expression level of immunostaining targeted protein was examined by use of AxioVision software (Carl Zeiss MicroImaging).

### Western blot analysis

Cells were harvested and washed with ice-cold PBS, and subsequently lysed in RIPA lysis buffer (Santa Cruz Biotechnology, USA). Protein concentration was measured by Pierce 660 nm protein assay (Thermo Scientific, Rockford, USA) according to the manufacturer's protocol. Protein samples from the treated cell lysates were separated by 10% or 6-12% SDS-PAGE, and further electrotransferred onto PVDF membranes (Millipore), which were then washed and blocked with 1× TBS containing 0.1% (v/v) Tween 20 and 5% (w/v) skimmed milk for 1 h at room temperature (RT). After washing, the membranes were incubated with the indicated primary antibody for at least 12 h, and further incubated with an appropriate horseradish peroxidase-conjugated secondary antibody at RT for 2 h. Visualization of reactive protein bands was performed using enhanced chemiluminescent detection reagents (Amersham; Thermo Scientific), and the expression level of reactive protein bands was further quantified using Image Studio Lite software (LI-COR Biosciences).

### Immunofluorescence cell staining

MDA-MB-231 cells (2 × 10^4^ cells/well) were plated on glass slips in 24-well culture plates and incubated overnight to allow cell adhesion, and then treated with vehicle (DMSO, 0.5%), DET (11 μM), DETD-35 (3 μM), or PTX (1 μM) for 24 h. The vehicle and compound treated cells were fixed with ice-cold 100% methanol for 10 min, and then permeabilized and blocked with blocking buffer (1× PBS containing 0.1% (v/v) Tween 20 and 3% (w/v) BSA) at RT for 1 h. After blocking, cells on glass slips were incubated with the indicated primary antibody in blocking buffer (1:100) at 4°C for 18 h, and then washed and stained with FITC conjugated secondary antibody (1:200) at RT for 3 h (Jackson ImmunoResearch Laboratories). The nuclei region of treated cells was stained with DAPI (Sigma-Aldrich), and the cells were further mounted onto a glass slide with Gold Antifade Reagent (ProLong). Visualization and capture of stained cells was accomplished through the Zeiss LSM 780 plus Elyra confocal microscope.

### Measurement of reactive oxygen species

To determine reactive oxygen species (ROS) level, treated cells were measured using the Total ROS Detection Kit according to the manufacturer's protocol (Enzo; Catalog No. 51011). The cells (1 × 10^5^ cells) were grown on 10-cm plates overnight and treated with vehicle (DMSO, 0.5%), DET (11 μM), DETD-35 (3 μM), or PTX (1 μM) for 1 h. Then, the cells were collected and resuspended in ROS detection solution (green probe) that directly reacts with a wide range of reactive species, for example hydroxyl radicals, hydrogen peroxide, peroxynitrite, peroxy radical, and nitric oxide, and the cells were further stained for 30 min at 37°C. After staining cells, the ROS level of treated cells was determined by flow cytometry.

### Transmission electron microscopy

The cells were prefixed with 0.1 M cacodylate buffer containing 2.5% glutaraldehyde and 0.1% tannic acid for 30 min at RT, and washed with PBS. Postfixing was performed by using 1% osmium tetroxide in 0.1 M cacodylate buffer at RT for 30 min, and then washed and dehydrated with graded series of ethanol (30-100%). After dehydration, the cells were embedded in Spurr's resin (EMS), and polymerized at 70°C for 48 h. The polymerized samples were sectioned and stained with uranyl acetate and lead citrate, and further examined using electron microscopy (FEI Tecnai G2 F20 S-TWIN FEGTEM).

### Transfection of small hairpin RNA

For knockdown of specific genes in cancer cells, the lentiviral-based small hairpin RNA (shRNA) clones employed in this study were purchased from the RNAi Core Facility, Academia Sinica. The cells (2 × 10^4^ cells/well) were plated in 12-well culture plates and incubated overnight to allow cell adhesion, and then replaced with fresh media containing polybrene and virus carrying shRNA targeting LC3, RAF1, or control (shLacZ) and then incubated overnight again. The LC3 and RAF1 shRNA sequences are as follows: 5′-CCGGCGCTTACAGCTCAATGCTAATCTCGAGATTAGCATTGAGCTGTAAGCGTTTTTTG-3′ for LC3; 5′-CCGGCATGAGTATTTAGAGGAAGTACTCGAGTACTTCCTCTA AATACTCATGTTTTT-3′ for RAF1. Selection of efficiently infected cells, the fresh media containing puromycin was used to incubate the cells for 48 h at 37°C.

### Statistical analysis

All data are presented as means ± standard deviation (SD). Statistical analysis of experimental data was performed by the SAS program (SAS Institute), and significant differences between different treatment groups were determined by ANOVA. P values of less than 0.05 were considered statistically significant.

## SUPPLEMENTARY MATERIALS FIGURES



## Abbreviations

DET: deoxyelephantopin
DETD-35: DET derivative-35
PTX: paclitaxel
ROS: reactive oxygen species
TNBC: triple negative breast cancer
ER: endoplasmic reticulum
LC3: microtubule-associated protein 1 light chain 3
MAPK: mitogen-activated protein kinase
ERK: extracellular signal-regulated kinase
JNK: c-Jun N-terminal kinase
RAF1: v-raf-1 murine leukemia viral oncogene homolog 1
shRNA: small hairpin RNA
CHX: cycloheximide
BAF-A1: bafilomycin A1
3-MA: 3-Methyladenine
NAC: *N*-acetyl-L-cysteine
GSH: reduced glutathione
