# Supplementary material for: Phytoagent deoxyelephantopin derivative inhibits triple negative breast cancer cell activity by inducing oxidative stress-mediated paraptosis-like cell death
Source: Oncotarget. 2017 May 25;8(34):56942–58. doi: 10.18632/oncotarget.18183 (PMC5593615; doi:10.18632/oncotarget.18183)
Supplement: Supplementary file 1 [file oncotarget-08-56942-s001.pdf]

Phytoagent deoxyelephantopin derivative inhibits triple negative breast cancer cell activity by inducing oxidative stress-mediated paraptosis-like cell death

SUPPLEMENTARY FIGURE

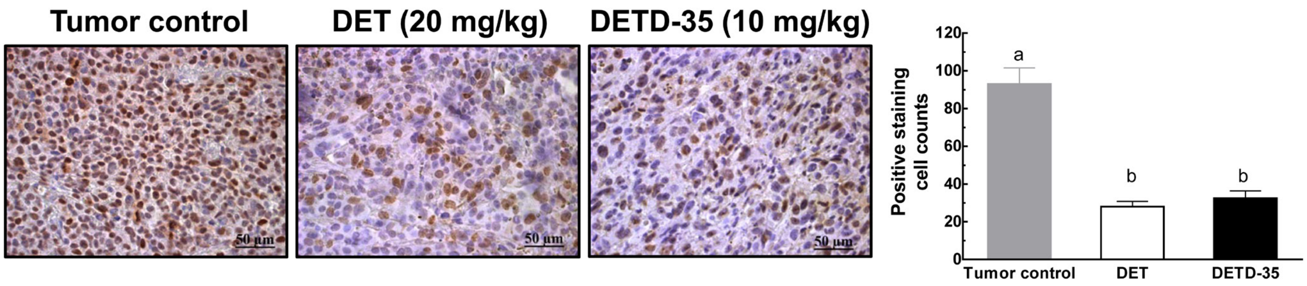

Supplementary Figure 1: Histological examination of proliferation marker Ki-67 expression in tumor tissues of mice with or without compound treatment. Representative immunohistochemistry images of tumor sections from each group for staining with Ki-67 and quantification of the Ki-67-positive staining cells from mouse tumor tissue sections of *in vivo* MDA-MB-231 xenograft study. Data are mean ± SEM, $n = 3$. Different letters represent significant differences (one-way ANOVA, $P < 0.05$).
